# The role of noncoding RNAs in Parkinson’s disease: biomarkers and associations with pathogenic pathways

**DOI:** 10.1186/s12929-021-00775-x

**Published:** 2021-11-18

**Authors:** Ming-Che Kuo, Sam Chi-Hao Liu, Ya-Fang Hsu, Ruey-Meei Wu

**Affiliations:** 1 Department of Medicine, Section of Neurology, Cancer Center, National Taiwan University Hospital, Taipei, Taiwan; 2 Department of Neurology, National Taiwan University Hospital, College of Medicine, National Taiwan University, Taipei, Taiwan; 3 Graduate Institute of Brain and Mind Sciences, College of Medicine, National Taiwan University, Taipei, Taiwan

**Keywords:** Noncoding RNA, Parkinson’s disease, MicroRNA/miRNA, PIWI-interacting RNA/piRNA, Circular RNA/circRNA, Long noncoding RNA/lncRNA, RNA sequencing, Biomarker

## Abstract

The discovery of various noncoding RNAs (ncRNAs) and their biological implications is a growing area in cell biology. Increasing evidence has revealed canonical and noncanonical functions of long and small ncRNAs, including microRNAs, long ncRNAs (lncRNAs), circular RNAs, PIWI-interacting RNAs, and tRNA-derived fragments. These ncRNAs have the ability to regulate gene expression and modify metabolic pathways. Thus, they may have important roles as diagnostic biomarkers or therapeutic targets in various diseases, including neurodegenerative disorders, especially Parkinson’s disease. Recently, through diverse sequencing technologies and a wide variety of bioinformatic analytical tools, such as reverse transcriptase quantitative PCR, microarrays, next-generation sequencing and long-read sequencing, numerous ncRNAs have been shown to be associated with neurodegenerative disorders, including Parkinson’s disease. In this review article, we will first introduce the biogenesis of different ncRNAs, including microRNAs, PIWI-interacting RNAs, circular RNAs, long noncoding RNAs, and tRNA-derived fragments. The pros and cons of the detection platforms of ncRNAs and the reproducibility of bioinformatic analytical tools will be discussed in the second part. Finally, the recent discovery of numerous PD-associated ncRNAs and their association with the diagnosis and pathophysiology of PD are reviewed, and microRNAs and long ncRNAs that are transported by exosomes in biofluids are particularly emphasized.

**Supplementary Information:**

The online version contains supplementary material available at 10.1186/s12929-021-00775-x.

## Background

After Alzheimer’s disease (AD), Parkinson’s disease (PD) is the second most common neurodegenerative disease worldwide. Gross pathology shows excessive loss of dopaminergic neurons in the substantia nigra (SN) at the midbrain and subsequent dopamine deficiency in the nerve terminals of the basal ganglia. Conventionally, if more than 50% of dopaminergic neurons are damaged [54], individuals show motor deficits such as bradykinesia (slow movement), limb rigidity, tremor at rest, gait disturbance (small stepped gait), and postural instability [147]. Currently, various brands of levodopa and dopamine agonists have been developed to alleviate motor symptoms, but none of them can slow the progression of the disease [55, 82]. Moreover, dopaminergic agents have little effect on controlling nonmotor symptoms of PD, such as anxiety, depression, sleep disorders, autonomic dysfunction, constipation, or cognitive decline [169]. The hallmark of the pathological findings of PD is the formation of eosinophilic inclusions called Lewy bodies (LBs) in the cytoplasm of dopaminergic neurons in the midbrain. In recent years, biochemical experiments have revealed the transformation of normal soluble α-syn monomers into pathological insoluble oligomer and fibril forms, resulting in large aggregates in the LBs. Increasing evidence suggests that these α-syn aggregates can spread from cell to cell through various transport routes [15, 64, 185] and extend remotely even from the gut to the brain via the highway-like vagus nerve or olfactory bulb [14].

In addition, α-syn pathology (synucleinopathy) is found in other central nervous system diseases. The deposition of α-syn in oligodendrocytes is the hallmark of multiple system atrophy (MSA), which is characterized by cerebellar ataxia, pyramidal signs, and autonomic dysfunction [53, 62]. Patients who suffer from dementia with Lewy bodies (DLB) were found to have early cognitive decline and prominent psychiatric symptoms such as hallucinations along with parkinsonism [122]. However, patients with tau-related pathological deposition in the central nervous system, termed tauopathy, also suffer from parkinsonism and other clinical manifestations, such as gaze palsy in progressive supranuclear palsy (PSP) [76, 205], or asymmetric parkinsonism and high cortical dysfunction in corticobasal degeneration (CBD) [6]. Those diseases that show atypical parkinsonism are overall classified as parkinsonism plus syndromes, as shown in Table 1 [206]. In clinical practice, how to precisely differentiate patients who have similar clinical features of parkinsonism is a major challenge to physicians. Hence, an objective, clear-cut and robust diagnostic biomarker of PD remains an unmet need. Although multiple biochemical and imaging biomarkers have been proposed for PD, none of them are convincing [107].

**Table 1:** The overview of Parkinson disease and parkinsonism plus syndrome

Disease	Pathological change	Major involved brain regions of Synucleinopathies	Levodopa response	Clinical features
PD	α-syn in neurons	Midbrain	Good	Asymmetric parkinsonism including rigidity, bradykinesia, or resting tremor
MSA	α-syn in oligodendrocytes	Cerebellum, striatum	Poor	Autonomic dysfunction, cerebellar ataxia, less asymmetric parkinsonism than PD
DLB	α-syn in neurons	Neocortex, limbic areas	Poor	Fluctuating consciousness and cognition, recurrent visual hallucination, symmetric parkinsonism
PSP	Tau in neurons	Brainstem, subcortex	Poor	Vertical supranuclear palsy, axial rigidity, cognitive impairment, symmetric parkinsonism, early fall
CBD	Tau in neurons and astrocytes	Frontoparietal cortex	Poor	Akinetic rigidity, limb apraxia, speech and language deficits, asymmetric parkinsonism, myoclonus, dystonia

*PD* Parkinsons’s disease, *MSA* multiple system atrophy, *PSP* progressive supranuclear palsy, *CBD* corticobasal degeneration, *DLB* dementia with Lewy bodies, *α-syn* alpha-synuclein

Recently, dysregulation of noncoding RNA (ncRNA) levels has been reported in several neurodegenerative disorders such as AD, PD and Huntington disease (HD) [179]. Although ncRNAs are not transcribed into proteins, ncRNAs still play several key roles in fundamental and complex biological processes such as development, inflammation, ageing, and degeneration [163]. Recent advances in sequencing technologies have further identified PD-associated ncRNAs and the function of ncRNAs in PD. In this review article, we will first introduce the biogenesis of different ncRNAs, including microRNAs (miRNAs), PIWI-interacting RNAs (piRNAs), circular RNAs (circRNAs), long noncoding RNAs (lncRNAs), and transfer RNA (tRNA)-derived fragments (tRFs). Then, we will discuss the pros and cons of the detection platforms and reproducibility of bioinformatic analytical tools due to their major impact on data consistency. In the final section, we will summarize the recent discovery of numerous PD-associated ncRNAs that may serve as novel biomarkers in the differential diagnosis of PD and play important roles in the pathophysiology of PD.

### Classification of noncoding RNAs

A substantial variety of ncRNAs have been uncovered in recent decades. In ncRNA history, transfer RNAs (tRNAs) and ribosomal RNAs (rRNAs) were first discovered in the 1950s and were also recognized as ncRNAs. The generic term ncRNA was not officially proposed until the discovery of small nuclear RNAs (snRNAs) and their neighbours, small nucleolar RNAs (snoRNAs), in the 1980s [20, 42]. However, in the early 2000s, the discovery of miRNAs finally attracted the attention of researchers worldwide because of their potential in orchestrating human gene expression mainly through post-transcriptional regulation [192]. Despite considerable scientific breakthroughs in the biological and medical aspects of miRNAs, our understanding of ncDNA continues to expand as ncRNAs are continuously identified. We recommend comprehensive reviews published in *Cell* in 2014 [27] and *J Biomed Sci* in 2020 [209]. Here, we focus on updated findings of miRNAs and recent studies introducing the biogenesis of novel categories of ncRNAs, including PIWI-interacting RNAs (piRNAs), circular RNAs (circRNAs), long noncoding RNAs (lncRNAs), and tRNA-derived fragments (tRFs), in the first section.

#### MicroRNA

MicroRNAs are small noncoding RNAs of approximately 21–25 nucleotides (nt) in length. The first miRNA, “lin-4 RNA”, was discovered in *Caenorhabditis elegans* and shown to participate in aberrant temporal regulation in the early developmental stage of *C. elegans* larvae [100, 204]. Since then, mounting evidence has demonstrated that miRNAs are expressed heterogeneously in different tissues [98] and translocate between subcellular compartments to mediate translational repression in numerous cellular processes, such as the regulation of cell differentiation, cell death and homeostasis [59].

**Fig. 1:**
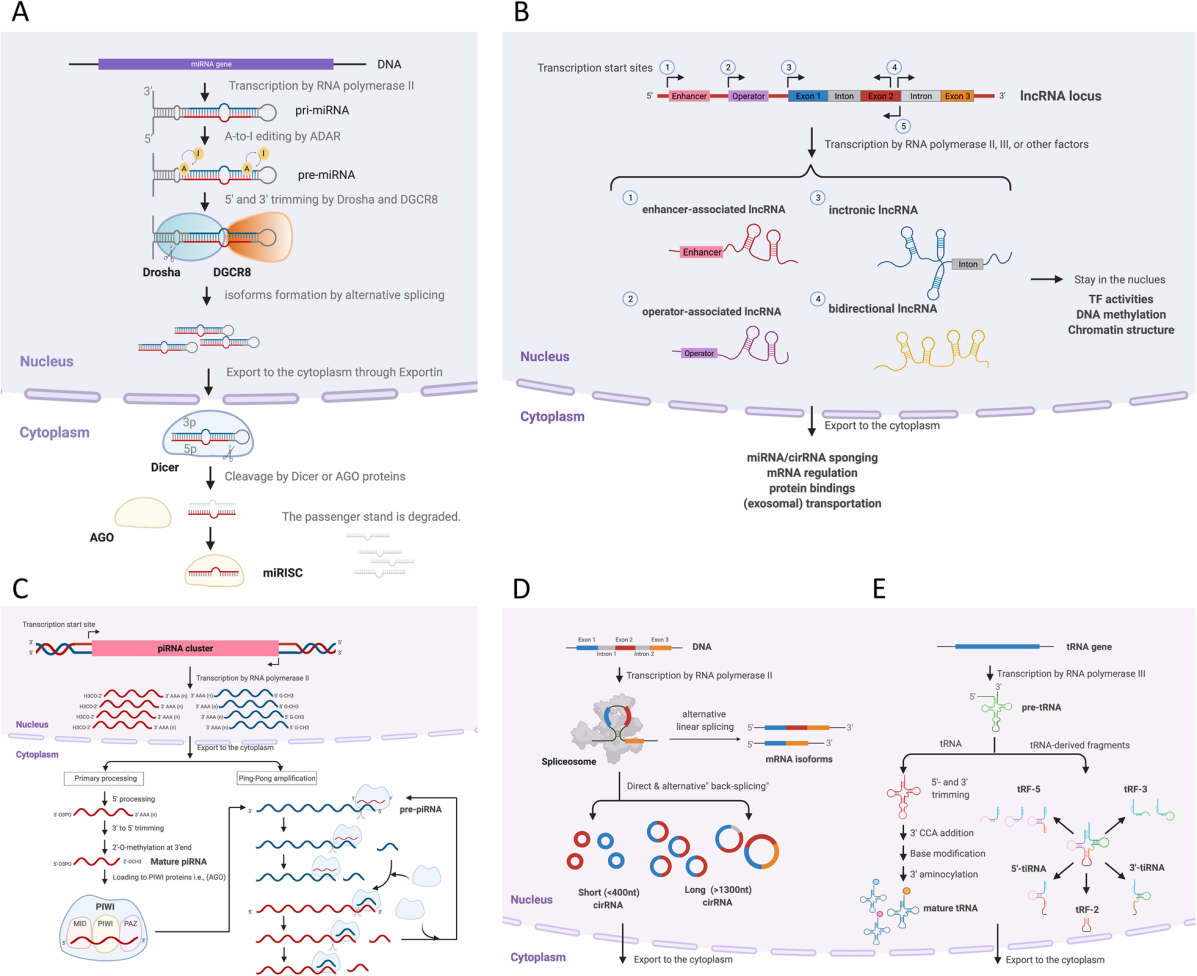
The biogenesis of miRNA, lncRNA, piRNA, circRNA and tRNA-derived fragments. **A** Several steps are required to produce mature miRNA, including A-to-I nucleotide editing by ADAR, 5’ and 3’ end trimming by Drosha and DGCR8, cleavage of double-stranded precursor miRNA (pre-miRNA) by Dicer through Dicer-dependent pathway or by AGO through Dicer-independent pathway, and finally, formation of miRNA-induced silencing complex (miRISC) with AGO proteins to bind other DNA or RNA targets. Some miRNAs will be modified by the adenosine deaminase (ADAR) proteins which act on double-stranded RNA to do A-to-I editing [148]. Next, two essential enzymes including protein DiGeorge syndrome critical region 8 (DGCR8) and Drosha forms the microprocessor complex and alternatively excise a pre-miRNA and remove the 5’ and 3’ terminals to produce a pre-miRNA duplex [105]. Pre-miRNA duplex in the nucleus is exported to the cytoplasm and is bound by Dicer to cleave the terminal loop to generate a mature miRNA duplex. Therefore, this process produces the isomirs, including 3p, 5p or polymorphic isomirs, which are classified by the arm of their pre-miRNAs [145]. **A** Genomes transcribed by RNA polymerase II (Pol II) produce mRNA but also lncRNA in both the sense and antisense directions. Most lncRNAs remain in the nucleus, while some of them are exported to the cytoplasm. **C** The piRNA clusters are a locus that can generate a family of piRNAs from the same single-stranded RNA transcript. PiRNA precursors are cleaved by PIWI proteins. Similar to the AGO protein in miRNAs, PIWI proteins also have three domains: MID, PIWI, and PAZ. **D** CircRNAs are preferentially generated by the noncanonical head-to-tail splicing of single or multiple exons, a mechanism called “alternative back-splicing (ABS)” by the depleted spliceosome. **E** Precursor tRNA (pre-tRNA) encoded from the tRNA gene is first trimmed by the endonucleases RNase P and RNase Z at its 5' and 3' ends, forming a cloverleaf-like tertiary structure composed of three stem loops (D loop, anticodon loop, and T loop), undergoing aminoacylation, and ultimately exporting out of the nucleus as a mature tRNA

The biogenesis of miRNAs comprises a series of processes, generally including canonical and noncanonical pathways [135]. Both pathways are sometimes closely interlinked and use the same factors. In brief, as shown in Fig. 1A, miRNA genes are first transcribed into primary miRNAs (pri-miRNAs) and processed into precursor miRNAs (pre-miRNAs) [96]. Both the 5' and 3' arms of pre-miRNA can generate functional mature miRNAs. The passenger strand of miRNA (termed miRNA-star or miRNA* was once thought to be useless and doomed to be degraded. More recently, both miRNA and miRNA* were shown to coexist and function [139, 216]. Additionally, the ratio of 5p and 3p strands was found to be tissue-specific in mice [157]. Hence, the nomenclature of miR-#-5p or miR-#-3p is recommended. Since the mechanism of miRNA strand selection is still unclear [124], the role of miRNA*s in gene regulation is still under debate, and thus, the physiological relevance of some passenger strands has been assumed to be underestimated [139]. How the cell decides or selects which miRNA strands to be dominant, called “arm switching”, requires further study [67, 124]. Owing to the complexity of nomenclature, readers should note which target miRNA is selected from previous studies when assessing the biological function.

In addition, the mechanisms by which miRNAs promote gene silencing through the inhibition of translation and the degradation of target mRNAs are continuously being discovered. Recent studies have focused on the mechanisms of miRNA-mediated post-transcriptional regulation [135], mostly by RNA binding proteins (RBPs) and other lncRNAs, the latter will be described in the next section. Since the regulation of miRNA function is still unclear and remains to be uncovered, we will not introduce all the cellular pathways and detailed mechanisms here. However, as miRNAs were also found to be secreted in extracellular fluids, probably through packing into exosomes [116], they have potential to serve as biomarkers for various diseases, including PD [80] (Table 2).

#### Long noncoding RNA

Similar to mRNAs in many ways, lncRNAs have a 5' terminal methyl guanosine (5'mG) cap and a 3' terminal polyadenylated tail, contain exons and introns, include several alternative splicing sites, and have no open reading frame (ORF) [145]. Genomes transcribed by RNA polymerase II (Pol II) produce mRNA but also lncRNA in both the sense and antisense directions (Fig. 1B). The length of lncRNAs is generally defined as longer than 200 nucleotides [77]. Most lncRNAs remain in the nucleus, while some of them are exported to the cytoplasm. Increasing evidence has shown the many biological functions of lncRNAs in cell biology, including direct DNA or mRNA binding, chromatin modifier regulation, post-transcriptional modification, and chromatin 3D structure formation, which have been thoroughly reviewed previously [183, 223].

Given that the mammalian brain contains up to 40% heavily transcribed lncRNAs [16], many aspects of lncRNAs are deeply involved in neuronal development and play a role in the pathophysiology of various neurological disorders, such as AD, PD, and HD [201]. Here, we briefly introduce lncRNAs and present the whole picture of the complex interplay of lncRNA-DNA and lncRNA-protein interactions and the complicated RNA machinery network, which is reviewed elsewhere [129]. Nonetheless, PD-related studies will be highlighted in the last section of this review.

#### PIWI-interacting RNA

Various researchers found a novel-at-the-time class of ncRNAs that is abundant in the fly [5] and mouse testes [3], later termed PIWI-interacting RNA (piRNA) [63]. A mature piRNA is typically 25–32 nucleotides in length after cleavage from a premature long sequence of a piRNA cluster. Increasing studies of piRNAs have revealed the delicate interactions of piRNAs with transposon elements (TEs), so-called jumping genes, which account for 90% of the repeated sequences (approximately 45% of the total genome) in noncoding regions and can stabilize the genome in a wide variety of species by regulating epigenetic modification and maintaining genome integrity [51]. Currently, our understanding of piRNA biology has substantially deepened. The piRNA clusters are a locus that can generate a family of piRNAs from the same single-stranded RNA transcript. Unlike miRNA processing with Dicer, piRNA precursors are cleaved by PIWI proteins. Similar to the AGO protein in miRNAs, PIWI proteins also have three domains: MID, PIWI, and PAZ (Fig. 1C). The difference between PIWI and AGO is that PIWI preferentially binds to the 3' terminal 2'-O-methyl group of piRNA, resulting in a closer link of PIWI to piRNA processing. PiRNAs can be roughly divided into two sets: one in gonad cells and another in somatic cells. Gonad-derived piRNAs typically follow the principle of “1U-10A” on their RNA transcripts, which enables these sets of piRNAs to undergo a unique self-amplification process named the “ping-pong cycle [4, 70] using transposon transcripts as templates. Notably, the unique 1U-10A template on the first 10th nucleotides of piRNA transcripts provides an opportunity to computerize and detect putative piRNAs by sequencing datasets. The second but relatively limited set of piRNAs is generated by “primary processing” of somatic cells.

In fact, the abundance of PIWI proteins and their homologues in animals, at least in pigs, is highest in the gonad, the kidney, and finally the brain [93]. Emerging data reveal the unexplored link of piRNAs in neural development, axonal regeneration, and memory formation, as described in a review [81], probably reflecting the fact that a set of TEs has a higher level in neuronal cells [128]. In recent decades, research on piRNAs has shifted from germline cell development to neurological disorders, including brain tumours [160], AD [151, 161], HD [49], and ageing, and this subject is increasing in popularity. These studies have only profiled piRNA identities in various neurological diseases. More issues merit a detailed investigation, including complete piRNA databases across various species and cells and the time- and space-specific expression pattern in tissues. All these works and valuable efforts can help elucidate aberrant piRNA biological networking in diseases with unmet medical needs, including PD.

#### Circular RNA

Circular RNAs were first reported as a plant viroid RNA structure in 1976 [164]. Typically, the eukaryotic proteome is translated from canonical linear mRNA transcribed by Pol II, while some isoforms of protein products can be generated by alternative splicing [133]. It has been proposed that when downstream mRNA processing is limited under certain conditions, circRNAs are preferentially generated by the noncanonical head-to-tail splicing of single or multiple exons, a mechanism called “alternative back-splicing (ABS)” by the depleted spliceosome [105] (Fig. 1D).

The first study describing the biological function of circRNAs in human brain development was published in 2013, proposing that a circRNA that is antisense to cerebellar degeneration-related protein 1 (CDR1as) can sponge many miRNAs, particularly miR-7, and therefore achieve post-translational gene regulation [125]. Since then, increasing evidence has shown that circRNAs are abundant in human tissues, particularly in the brain [162]. Furthermore, a comprehensive analysis revealed that circRNAs are highly enriched in the frontal cortex, hippocampus, and cerebellum [65], and the expression of circRNAs is highly spatially and temporally dynamic in the central nervous system (CNS) [230]. Accumulating data have promoted interest in studying the transcriptomic regulatory networks of circRNAs in various neuropsychiatric disorders [115]. To date, most circRNAs are being studied in the field of cancer [217], with some in cardiovascular diseases or dementia, but these molecules have rarely been explored in the field of PD. For more information on the functional impact of circRNAs, some comprehensive reviews are worth reading [30, 214].

#### Transfer RNA-derived fragments

Transfer RNA is transcribed by RNA polymerase III and highly abundant, accounting for up to 15% of all RNA in tissues and cells [144]. Precursor tRNA (pre-tRNA) encoded from the tRNA gene is first trimmed by the endonucleases RNase P and RNase Z at its 5' and 3' ends, forming a cloverleaf-like tertiary structure composed of three stem loops (D loop, anticodon loop, and T loop), undergoing aminoacylation, and ultimately exporting out of the nucleus as a mature tRNA (Fig. 1E). The demographic summary in a recent review is worth reading [186]. After export out of the nucleus, tRNA canonically pairs triplet anticodons with the complementary codon on mRNA and facilitates peptide elongation and protein translation.

Accumulating evidence has revealed that fragments of mature and precursor tRNAs, including tRFs and tiRNAs, once considered redundant in cell biology, have additional noncanonical functions, such as miRNA gene silencing, post-translational modification of mRNA, protein interactions, epigenetic modification, cellular stress responses, and sperm maturation [172]. However, most studies on disease-relevant tRNA-derived fragments are in the field of cancer. We refer readers to some excellent reviews [211]. In PD, tRFs are still a growing field waiting to be explored.

### Techniques for noncoding RNA detection and analysis

Total RNA, when originally isolated, is composed of multiple RNA species, including rRNA, precursor messenger RNA (pre-mRNA), messenger RNA (mRNA), and several types of ncRNAs. Noncoding RNAs are highly heterogeneous in terms of their length and conformation. These molecules can be separated into 3 categories: (1) small ncRNAs (< 50 nt), including microRNAs (miRNAs; 19–25 nt), small interfering RNAs (19–29 nt), PIWI-interacting RNAs (piRNAs, 25–31 nt), and other functional small RNAs, such as transcription initiation RNAs (tiRNAs, 17–18 nt), tRNA-derived fragments (tRFs, 14–36 nt), snoRNA-derived RNAs (17–24 nt or > 27 nt), and sectional ribosomal RNA-derived fragments (rRFs, 15–81 nt); (2) intermediate-sized ncRNAs (50–500 nt), including 5S rRNAs (~ 120 nt), 5.8S rRNA (~ 150 nt), tRNAs (76–90 nt), snoRNAs (60–300 nt), and small nuclear RNAs (snRNAs, ~ 150 nt); and (3) long noncoding transcripts greater than 500 nt, including linear lncRNAs and circular circRNAs [189]. Over 90% of the total RNA molecules present in a cell are rRNAs and tRNAs, while small RNAs account for ∼1% or less [235]. These ncRNAs can regulate gene expression by directly or indirectly binding to specific DNA or RNA sequences.

Because many of these ncRNAs are tissue- and disease-specific, the expression profile (the RNA sequences and expression levels) of these ncRNAs can be used as biomarkers for diseases. Different platforms can provide different information about the sequence and expression level of ncRNAs. Sometimes a combination of these platforms could be used to obtain a more comprehensive view of the expression profiles of multiple RNAs. In this part of the review, the basic principle of five common platforms, microarray, RT-qPCR, Illumina NGS, PacBio and Nanopore, will be discussed. We will focus on how each platform can be used to generate the expression profiles of ncRNAs, including known and unknown transcripts. Non-coding RNAs include small RNAs (miRNAs, piRNAs and tRFs) and long RNAs (lncRNAs and circRNA).

### Small ncRNAs

When total RNA is extracted from biological materials (e.g., cells or tissues), subsets of RNA molecules need to be isolated or enriched using specific protocol, such as a ribodepletion protocol to remote ribosomal RNAs or size selection by electrophoresis, to filter out unwanted transcripts [97]. RNA isolation and library preparation strongly affect the detection of target species of ncRNAs [189].

With a mixture of small RNAs, since miRNAs (19–25 nt), piRNAs (25–31 nt), and tRFs (14–36 nt) are all similar in size, they cannot be easily distinguished by examining their sizes. Luckily, most of the time these small RNAs could be identified in the database that contains previously reported transcripts. In some cases when the database or reference sequences are not available, specific characteristics of miRNAs, piRNAs and tRFs need to be considered to aid the identification of the novel transcripts.

#### Characteristics of miRNAs, piRNAs and tRFs

Typical miRNAs have the following characteristics: (1) their length distribution is narrow and often between 21 and 23 nt; (2) their precursor forms a hairpin structure; therefore, the genomic sequencing flanking a miRNA sequence contains a highly complementary 20- to 30-nt segment; (3) in most cases, pre-miRNA processing results in asymmetric strand accumulation; (4) a miRNA 5' end is most often uridine; (5) a 3' end is usually variable and, at low frequency, can be post-transcriptionally modified by the addition of adenosine or uridine; and (6) mature miRNAs, and often pre-miRNA sequences, are often conserved in closely related species; (7) in most cases, miRNAs originate from nonrepetitive genomic sequences [95]. During tRNA maturation, the 3′- trailer sequences are removed from pre-tRNA, which results in the production of 1-tRF [60]. The other two classes of tRFs are generated from mature tRNAs: 5′-tRF as produced by cleavage of the 5′ end in the D-loop and 3′- tRF as produced through cleavage of the 3′ end in the T-loop [227]. Since tRFs are fragments of tRNAs, a tRF should partially resemble to the tRNA sequence [196]. There is no common features for all piRNAs yet. Therefore multiple features if often employed when identifying piRNA. For example, several K-mers based features had been proposed [109]. Some position-specific properties include a preference for uridine at the 5′ end (75.81%), which is a main characteristic of primary piRNAs, and an A-bias at the 10th nucleotide position (40.61%) [40]. Since there is no consistent characteristics of all piRNAs, the analysis of piRNAs is often done after the depletion of other types of small RNAs [108].

#### Microarray

To determine the expression profile of these small RNAs, researchers can use different platforms. Microarray is often chosen if the sequences of the sample are known and we want to determine the differential expression.

**Fig. 2:**
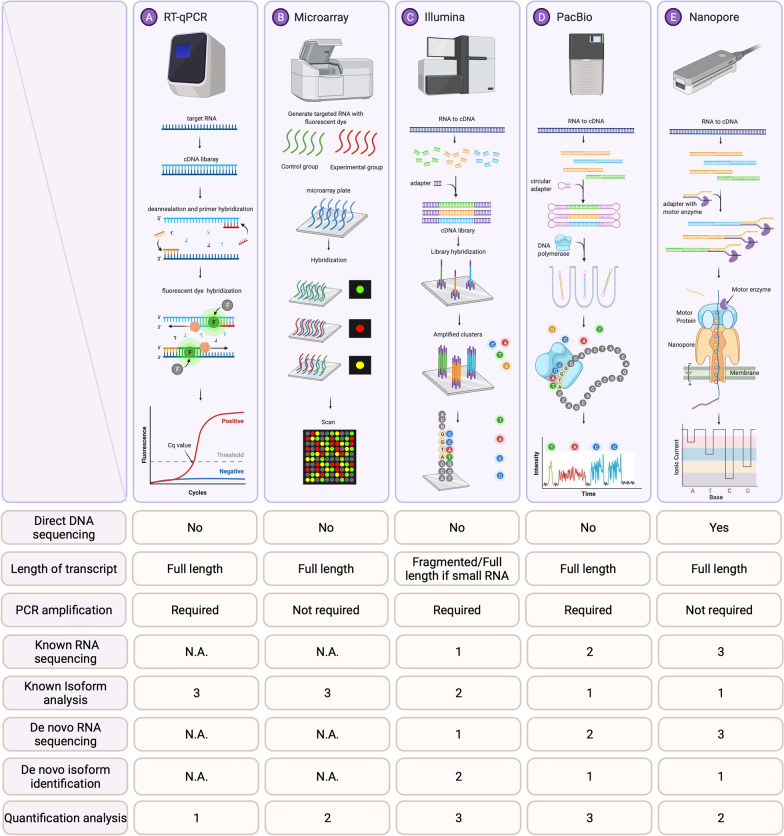
RNA sequencing technologies and workflows of qPCR, microarray, Illumina NGS, PacBio and ONT (Oxford Nanopore Technology). **A** cDNA is synthesized by reverse transcription of extracted RNAs. Fluorescent signals are emitted and detected by the qPCR instruments during PCR. The figure shows the basic principle of SYBR green detection. The quantitative real-time PCR amplification plot is shown at the bottom. The number of PCR cycles is shown on the x-axis, and the fluorescence from the amplification reaction, which is proportional to the amount of amplified product in the tube, is shown on the y-axis. **B** After the extracted RNA targets are converted to cDNA /cRNA templates, they can hybridize with the cDNA probes on the chips. The cDNA / cRNA samples would carry signals and are labeled with fluorescent tags. When target cDNA / cRNA templates bind to the complementary oligonucleotide probes, the signals will be released. The signal intensities correspond to the abundance of cDNA / cRNA binds to the probes. Each dot observed in the bottom figure represents a cumulative hybridization reaction. Red color denotes higher expression levels of experimental groups while green color denotes higher expression levels of the control group. cDNA arrays typically involve two channels (two colors in the **B**), but single channel (one color) is also available [178]*.* **C** After cDNA library preparation, individual cDNA molecules are clustered on a flow cell. Illumina NGS detects the sequences by synthesis using fluorescent labelled nucleotides. In each small step of sequencing, the growing DNA strand will emit signals from one of the four fluorophores when the nucleotide has been incorporated (images are modified from [202]. The emission wavelength and intensity are used to identify the bases. D After PacBio SMRT-adaptor ligation, circularized cDNA molecule is formed. The individual molecules are loaded into a sequencing chip, where they bind to a polymerase immobilized at the bottom of a nanowell. As each of the fluorescently labelled nucleotides is incorporated into the growing strand, the fluorescent signals are emitted and detected by the PacBio instrument (images are modified from [202]. cDNA sequencing on the PacBio platform enables full-length sequencing from 5’ cap to the 3’ RNA cleavage site. (E) After library preparation, individual molecules attached with motor protein during adaptor ligation are loaded into a flow cell. The motor protein controls the translocation of the RNA strand through the nanopore, causing a change in current that is characteristic for the subsequent bases and will serve as the basis for basecalling. The figure at the bottom shows the corresponding electrical currents (in the pA-range) to nucleotides. The pros and cons of all platforms are listed in the Additional file 1. The numbers of 1, 2, 3 correspond to very good, good and fair performance of each platform. *N.A.* non-available

Microarrays are a major high-throughput (reads per run) tool that can simultaneously provide the relative concentration or expression levels of hundreds of target RNA templates [18] based on the computed scores of image intensity. The array platform consists of antisense DNA oligonucleotide probes, spotted or printed onto glass slides or nylon membranes, are designed to hybridize with the appropriate RNAs (small or long RNAs) [95]. Microarray data can immediately identify target molecules with up- or downregulated expression. Nonetheless, array technology has several limitations. For example, background hybridization limits the accuracy of expression measurements, particularly for transcripts present in low abundance. Furthermore, cross-hybridization, nonspecific hybridization and limited detection range of individual probes may occur. There are also issues associated with probe redundancy and annotation [233]. Specificity and sensitivity can be compromised in small RNAs with high sequence similarity due to the nature of short hybridization sequences [177]. Array hybridization methods normally require relatively large amounts (micrograms) of RNA therefore not sufficiently sensitive to profile miRNA expression in a single cell or few cells. Theoretically, reverse transcription quantitative real-time PCR (RT-qPCR) can provide a more sensitive method of profiling [95]. For the pros and cons of microarray, please refer to Fig. 2 and Additional file 1.

#### RT-qPCR

RT-qPCR is another platform that has often been used to analyse RNA or DNA expression profiles. Because RT-qPCR can provide high sensitivity and specificity for transcripts with various sizes, it is suitable for quantitative study. This method is also widely used in most research labs and by researchers. In fact, RT-qPCR is currently the gold standard method to verify data obtained by microarrays or next generation sequencing (NGS) approaches [177]. However, several drawbacks exist. Because primers are required for RT-qPCR, nonspecific primer design, inconsistent data analysis and normalization can negatively affect the reproducibility of this method [177]. Like microarrays, RT-qPCR can only interrogate a limited set of variants with known sequences and has limited discovery power. The capacity for the number of reactions it can perform each time is also limited. For the pros and cons of RT-qPCR, please refer to Fig. 2 and Additional file 1.

#### Illumina Next Generation Sequencing (NGS)

NGS is a good tool if we do not know the sequence of the target RNAs or want to study minor mutations or polymorphisms. We will focus on Illumina NGS since it is the major NGS platform currently in the market. NGS has been widely tested and used by many lapidaries, and the bias and error profiles are well understood. A large catalogue of compatible methods and computational workflows is also available. RNA-seq has considerable advantages for examining transcriptome fine structure, such as the detection of novel transcripts, allele-specific expression and splice junctions [233].

NGS is a very high throughput (reads per run) platform and currently provides 100–1000 times more reads per run than long-read platforms. NGS can not only profile the expression of known RNA sequences but is also suitable for identifying various unknown variants [177] and can read degraded or truncated RNA. These fragments or short transcripts will be turned into cDNA libraries through cDNA synthesis. Fragmentation is not required when sequencing small RNAs. Because each fragment can be sequenced repeatedly, NGS has the highest accuracy in detecting the sequence of the transcripts. Low error rates are particularly important for sequencing miRNAs, whose relatively small sizes could result in misalignment or loss of reads if error rates are too high. Because of its high throughput, this method is also suitable for minor variant detection, human axon and genomic sequencing, genome-wide association studies, and gene expression studies [2]. For detection of RNA transcripts with moderate to high abundance, 30–40 million reads are required to accurately quantify their expressions. For coverage over complex transcript libraries, including rare and lowly expressed transcripts, up to 500 million reads are required [56]. The unbiased data acquisition, sequence coverage and depths of NGS are unparalleled by any other available method, and this technique is the only discovery-based approach allowing the identification of novel small RNAs [177].

RNA-seq analysis is vulnerable to the general biases and errors inherent in the NGS technology. Because library preparation includes sequence fragmentation, adaptor ligation, cDNA synthesis and PCR amplification, errors can occur [175]. Uneven read coverage, complex splicing and potential sequencing bias could complicate the sequencing even more [138, 233]. The fragments are not uniformly sampled and sequenced, as there is variability in sequencing depth across the transcriptome due to preferential sites of fragmentation and variable primer and tag nucleotide composition effects [73, 121]. For the pros and cons of Illumina NGS, please refer to Fig. 2 and Additional file 1.

#### Expression profile by NGS

The expression level of each RNA unit is measured by the number of sequenced fragments that map to the reference transcript, which is expected to correlate directly with its abundance level. RNA-seq data (counts of mapped reads) are fundamentally different from microarray data (computed scores of image intensity) in terms of the expression level analysis. In RNA-seq, the expression signal of a transcript is dependent on the sequencing depth and the expression levels of other transcripts, whereas in array-based methods, probe intensities are independent of each other [153]. Normalized RNA-seq data, therefore, must be modeled statistically using discrete distributions.

The nature of the features encompassed by read count data depends on what the mapping reference (database) is and what type of aligner tool was used. Some types of analytical software are designed to handle isoform differences [101, 191], and others analyse generic features of the transcriptome [153]. To accurately estimate gene expression, read counts must be normalized to correct for systematic variability, such as library fragment size and read depth [97, 140, 158]. This issue, as well as other technical differences, has motivated the development of a growing number of statistical algorithms that implement a variety of approaches for normalization and differential expression (DE) detection [153].

### Long RNAs

There are currently three main platforms for long RNA sequencing and expression profiling: Pacific Biosciences (PacBio) and Oxford Nanopore Technologies (ONT). For long RNA sequencing, including lncRNA and circRNA sequencing, similar to short-read RNA sequencing, the target ncRNAs will firstly be specifically enriched as much as possible to reduce interference signals from other transcripts [66, 189]. Library preparation is first dependent on rRNA depletion methods, then reverse transcription (RT) with random primers and size selection by gel electrophoresis, leading to the deficiency of small ncRNAs [193]. Depletion of linear RNAs can be done by RNase R treatment, leading to the enrichment of circRNAs.

#### Characteristics of lncRNA and circRNA

Like small RNAs, databases are available for most of the lncRNAs and circRNAs. If certain lncRNAs or circRNAs cannot be found in the database, specific characteristics of lncRNAs and circRNAs should be considered to aid the identification of novel lncRNAs and circRNAs.

CircRNAs are a type of long RNA formed by covalent binding of the 3′ and 5′ ends after reverse splicing [207, 208]. The junction between two related exons in the opposite order is called the back-sliced junction (BSJ), which represent a molecular signature of circRNA [28, 57]. Many tools recognize circRNA by identifying the BSJ read. Most algorithms embedded in tools are based on splitting the reads (reads spanning BSJs are split into segments and are aligned to the reference sequence in reverse order) (called segmented-read-based), while several other tools are based on a pre-defined BSJ and flanking sequence of a circRNA (pseudo-sequences to recognize BSJ reads). These tools then map the read directly to that pseudo-reference for discovering a BSJ [28, 31, 57].

LncRNAs are non-coding RNAs with lengths greater than 200 nt [207, 208]. LncRNAs can be preliminarily defined as long RNA transcripts that are capable of autonomous transcription and are longer than 200 nt but lack the capacity to encode proteins [152]. The prediction of new lncRNAs includes two steps: basic screening and potential coding ability screening. Transcript lengths, exon numbers, ORF length and expression level are considered in the basic screening step [218]. Next, all transcripts with protein-coding potential are filtered out (CPC, CNCI, CPAT and Pfam software) [83].

#### NGS

Although microarrays and RT-qPCR can be used in long RNA expression analysis [102], since the primer or probe binding region is relatively short in the full-length transcript, some information, such as minor mutations, isoforms and structural variances, can be overlooked. NGS can also be used to determine the expression profiles of long RNAs. One major benefit of ensemble-based platforms if low sequencing error rates (< 1%) dominated by single mismatches [ref]. The long RNAs are fragmented, reverse transcribed into cDNA by random primers, and undergo end repair, sequencing adaptor ligation, and size selection for subsequent sequencing. In this way, not only lncRNAs but also mRNAs, circRNAs, and some intermediate-sized ncRNAs can be analysed by NGS. However, the reconstruction of transcripts from short-read data is difficult. Short RNA-seq reads capture only small fragments of transcripts. RNA-seq data, therefore, lack clear isoform data, leading to the inference of many erroneous isoforms. Most algorithms can identify discrete transcript components, but the assembly of complete transcript structures remains a major challenge [97, 184]. On the other hand, long-read full-length cDNA captures transcripts end-to-end, making isoform inference unambiguous [21].

#### PacBio

The two main long-read platforms are PacBio and Oxford Nanopore Technology (ONT). Long-read sequencing is more accurate and sensitive in structural variation and isoform detection [1]. Because it loads full-length cDNA sequences, this technique can capture many full-length transcripts (1–50 kb), including new isoform structural variations. Because reading results do not need to be reassembled, the computational methods for de novo transcriptome analysis are simplified. These methods are also suitable for studies of minor variations, novel assembly, epigenetics, and RNA isoforms [2]. However, the technology features low to medium throughput: currently, only 500,000 to 10 million reads per run (NGS obtains 100–1000 times more reads per run) [182]. Thus, this method requires high throughput to reach the same accuracy in terms of short-scale variations or low-frequency detections.

PacBio single-molecule real-time (SMRT) isoform sequencing (Iso-Seq) can capture the full length of transcripts, thereby presenting an easier and more accurate method for gene annotation, isoform identification, and lncRNA discovery [39]. Due to the large amount of template required for PacBio sequencing, large-volume PCR is performed. cDNA size selection is optional but highly recommended, as PacBio has weak power in detecting short RNA sequences or degraded RNA templates. After PCR end repair and PacBio SMRT-adaptor ligation, long-read sequencing is performed [182]. Another advantage of this sequencing approach is the ability to produce extraordinarily long reads (average lengths of 4200 to 8500 bp), which greatly improves the identification of novel transcript structures [7, 97, 173]. The extremely long reads generated by the PacBio platform are ideal for de novo transcriptome assembly in which the reads are not aligned to a reference transcriptome. Longer reads can facilitate the accurate detection of alternative splice isoforms, which may not be discovered with shorter reads.

The limitation of lower throughput and higher error rate is most obvious when performing the large-scale differential expression analysis. In these studies, the expression of ncRNA needs to be precisely profiled to attain sufficient statistical power to have confidence in the transcriptome fold changes. For the pros and cons of PacBio, please refer to Fig. 2 and Additional file 1.

#### Oxford Nanopore Technology (ONT)

ONT sequencers measure changes in ionic current when the DNA fragments translocate through protein nanopores in a semisynthetic insulated membrane; this process does not require enzyme-based nucleotide incorporation or detection of fluorescence signals [39].

Since PCR amplification is optional and direct cDNA sequencing is possible, some errors during DNA polymerase could be avoided (e.g., incorrect nucleotides might be added to the sequence during polymerase), leading to higher-quality results. However, the sequencing yields (numbers of reads) were higher for PCR-amplified cDNA libraries. If PCR is performed, users could start with much smaller amounts of input RNA [182]. Similar to that of PacBio, because it does not go through RNA fragmentation, the structure of the noncoding RNA is retained and can avoid computational algorithm error during template assembly. Thus, this method is suitable for detecting structural variations and isoforms (e.g., [188]. Because ONT can process cRNA directly without cDNA synthesis, RNA modification and epigenetic information are also retained, similar to methylation and other modifications [1, 182]. Expression is directly approximated by the number of reads that mapped on a given transcript [170]. Oikonomopoulos et al. [137] suggested that the expression levels of long RNAs are better captured by ONT, while cDNA-seq (Illumina) appears to be more biased. In general, ONT still has higher sequencing error rate than NGS and PacBio [202]. For the pros and cons of ONT, please refer to Fig. 2 and Additional file 1.

#### PacBio vs. ONT

For PacBio and ONT Pc data, we found that short read lengths (< 500 bp) had low alignment rates. This finding is likely due to a larger portion of adapter and linker sequences in this short-length data bin [39]. In practice, PacBio and ONT sequencing have their own merits and demerits [39]. One of the greatest advantages of ONT compared to PacBio sequencing is that it can estimate transcript expression levels [168]. In the present study, Cui et al. [39] analysed the correlation between Illumina and ONT data of each replicate sample and found correlations > 0.8 for all groups. The high correlation suggests that ONT can quantify transcript expression levels well. Briefly, PacBio was superior in identifying alternative splicing events, whereas ONT Pc could estimate transcript expression levels [39].

PacBio technology is now widely used for the characterization of cancer transcriptomes, where detection of novel isoforms and fusion transcript is superior to that of short-read technologies. This approach surpasses mapping-based or assembly-based approaches. The effectiveness of MinION in the accurate quantification of transcripts, in the detection of transcript variants and fused genes, in transcript-based haplotype phasing and allele-specific expression and in single-cell expression profiling has been shown in multiple studies [168]. In addition, full-length transcript sequence information is very useful for both genome annotation and gene function studies [137].

#### Brief Summary

Each platform has its strengths and weaknesses and can be used to address different research questions in RNA expression profiling. There is currently no one-size-fits-all approach for all aspects of RNA profiling. Microarrays and RT-qPCR provide direct quantification of target RNAs, but they are unable to identify novel RNA sequences and can only work with known sequences. Long-read sequencing platforms (such as PacBio and ONT) can be used to detect and analyse complete full-length RNA sequences and identify new RNA transcripts, structural variations and RNA isoforms [2], but because they have a higher error rate than short-sequencing platforms (such as NGS), they are not suitable for analysis that requires accurate sequencing, e.g., expression profiling. This method is not effective for analysis of truncated, degraded or small RNA transcripts. Short-read sequencing can provide indirect quantification, can identify novel RNAs, has high throughput (reads per run), and has many analytical tools. However, it is less accurate in analysing structural variations, RNA isoforms and comprehensive RNA transcriptomes [1]. Most (> 90%) ncRNAs are miRNAs, and in conclusion, different platforms are suitable for different levels of specificity and sensitivity and are often complementary to each other. For comprehensive noncoding RNA profiling, a combination of methods is recommended.

#### The role of noncoding RNAs in PD

Recent advances in sequencing technologies have enabled high-resolution RNA profiling and thus has provided insights into PD-associated ncRNAs. In the final section, the role of each RNA (miRNA, lncRNA, circRNA, pRNA and tRNA) in serving as potential biomarkers for PD is summarized. Their functional aspects in the pathogenesis of PD are also discussed separately.

### MiRNAs in PD

Many studies and review articles emphasize the orchestrated role of miRNAs in the pathogenesis, differential diagnosis, and prognosis of PD. Previous studies of microRNAs associated with PD are summarized in Table 2. A recent meta-analysis thoroughly summarized the identities of differentially expressed miRNAs in PD [165]. Theoretically, an ideal study design requires large-scale, longitudinal, international cohorts using multidisciplinary methods, including NGS, microarray, and RT-qPCR, to detect new types of ncRNAs, validate the regulatory direction of differentially expressed ncRNAs, and succeed in investigating their biological functions in the pathogenesis or prognosis of PD in translational studies. Kern at el. recently provided a good example that fulfils almost all these requirements [87]. These researchers reported circulating small ncRNA profiles, primarily (~ 93%) miRNAs, in two large-scale longitudinal cohorts (Parkinson’s Progression Markers Initiative (PPMI) and Luxembourg Parkinson’s Study (NCER-PD)) using NGS and microarray datasets, respectively. Comparing total PD groups (genetic and idiopathic) and control groups (healthy and unaffected) with some cases that were serially followed, they first identified both diagnostic and prognostic miRNA biomarkers in the PPMI cohort. Between total PD and controls, five miRNAs were differentially expressed in PD (upregulated expression of miR-6836-3p and miR-6777-3p and downregulated expression of miR-487b-3p, miR-493-5p, and miR-15b-5p). Only miR-15b-5p was differentially expressed in plasma-derived exosomes from PD patients [210], and the other 4 differentially expressed miRNAs have never been identified in PD before. Moreover, only downregulated expression of miR-487-3p and miR-15b-5p was validated between the PPMI and NCER-PD cohorts. A similar study was performed by the same group in a smaller cohort (106 PD patients versus 91 healthy controls (HCs)) [43], while 5 differentially expressed miRNAs at that time were not able to be reproduced in this larger cohort. The differences between the two studies, including sample size (ten times larger in Kern et al.’s study, sequencing platform (Illumina Solexa by Ding et al. NextSeq by Kern et al. and validated platform (microarray by Ding et al. RT-qPCR by Kern et al. may be responsible for the discrepancy between the two studies. Regarding prognostic miRNAs correlated with the worsening of motor symptoms and accompanied by the network analysis of miRNA-to-mRNA interactions, a total of 8 miRNAs with downregulated expression (let-7b-5p, miR-140-3p, miR-574-5p, miR-769-5p, miR-3157-3p, miR-3960, miR-5690, miR-6734-5p were highlighted in patients with a progressive course. However, the progression of other nonmotor symptoms, such as cognitive impairment or psychiatric symptoms, should also be important. Last, most of the participants in the PPMIR and NCER-PD cohorts were Caucasians, hindering universal application worldwide. This large-scale study noted the difficulties we found in PD-associated ncRNA studies, including lack of standard sequencing methods, few reproducible targets, and lack of multiethnic populations. The use of other sources of samples (e.g., blood, urine, or saliva,free circulating form or extracellular vesicle-derived form and enrichment of miRNA origins (e.g., brain or other organs; neurons or other neuronal cell types would have a greater impact on the final list of differentially expressed miRNA profiles. In fact, miRNAs could be further categorized according to their biological pathways associated with mitochondria, autophagy, inflammation, and PD-related genes, including *SNCA*, the gene encoding alpha-synuclein. Herein, we highlight some novel and innovative mechanisms of miRNAs that will shed light on future works in this section.

**Table 2:** The miRNA profiles associated with PD patients

Samples	Analysis methods	miRNA expression—upregulated	miRNA expression—downregulated	Description	Reference
Human Plasma	rt-qPCR	miR-27a	Let-7a, let-7f, miR-142-3p, miR-222	25 PD, 25 HC	[29]
	rt-qPCR	–	MiR-1, miR-22*, miR-29a	15 PD, 8 HC	[118]
	rt-qPCR, exosome	ex-miR-331-5p	ex-miR-505	52 PD, 48 HC	[224]
	rt-qPCR	miR-7-5p, miR-22-3p, miR-124-3p, miR- 136-3p, miR-139-5p, miR-330-5p, miR-433-3p, miR-495-3p	–	99 (idiopathic) PD, 101 HC	[154]
	rt-qPCR	miR-22-3p, miR-139-5p, miR-154-5p, miR-330-5p	–	109 PD, 92 HC	[155]
	rt-qPCR	miR-105-5p	–	317 PD, 273 HC	[220]
	rt-qPCR	miR-132	–	46 PD	[221]
	rt-qPCR	miR-331-5p	–	31 PD, 25 HC	[25]
	NGS + rt-qPCR	miR-338-3p, miR-30e-3p, miR-30a-3p	miR-16–2-3p, miR-1294	50 PD, 65 HC, (53 AD); serum + CSF;	[19]
	rt-Qpcr exosome	ex-miR-30c-2-3p	ex-miR-15b-5p, ex-miR-106b-3p,ex-miR-138-5p, ex-miR-338-3p	30 PD, 30 HC	[210]
	rt-qPCR	miR-137	miR-124	60 PD, 60 HC	[103]
	rt-qPCR, exosome	ex-miR-34a-5p	–	15 PD, 14 HC	[68]
	rt-qPCR	miR-30a-5p	–	(Set 1) 50 PD, 50 HC; (Set 2) 49 PD, 49 HC; meta-analysis for both set 1 and set 2	[167]
Human Serum	rt-qPCR	–	miR-132-3p, miR-146-5p	82 PD, 44 HC	[176]
	rt-qPCR	miR-29c	–	51 PD, 20 HC	[141]
	rt-qPCR	miR-30c-5p, miR-373	–	148 PD, 126 HC	[228]
	rt-qPCR	–	miR-29a, miR-29b, miR-29c	80 PD, 80 HC	[9]
	rt-qPCR, exosome	ex-miR-24, ex-miR-195	ex-miR-19b	109 PD, 43 HC	[24]
	rt-qPCR	let-7d, miR-22*, miR-23a, miR-24, miR-142-3p, miR-222	–	30 PD, 30 HC	[11]
	rt-qPCR	–	miR-29c, miR-146a, miR-214, miR-221	138 PD, 112 HC	[111]
	NGS (Illumina Solexa seq) + rt-qPCR	miR-195	miR-15b, miR-181a, miR-185, miR-221	106 PD, 91 HC	[43]
	NGS (Illumina Solexa seq) + rt-qPCR	–	miR-141, miR-146b-5p, miR-193a-3p, miR-214	169 PD, 180 HC	[44]
	NGS (Illumina NextSeq in PPMI cohort) + microarray (in NCER-PD cohort)	miR-6836-3p, miR-6777-3p	miR-487b-3p, miR-493-5p, miR-15b-5p	total 1614 in PPMI cohort; 440 PD, 485 HC in NCER-PD cohort	[87]
Human PBMCs	Microarray + rt-qPCR	–	miR-335, miR-374a, miR-199a-3p/miR-199b-3p, miR-126*, miR-151-3p, miR-199a-5p, miR-151-5p, miR-126, miR-29b, miR-147, miR-28-5p, miR-30b, miR-374b, miR-19b, miR-30c, miR-29c, miR-301a, miR-26a	19 PD, 13 HC; only miR-126* was validated by rt-qPCR	[120]
	rt-qPCR	miR-155-5p	miR-146a-5p	37 PD, 43 HC	[22]
Human leukocytes	NGS + rt-qPCR	miR-199b, miR-1274b, miR-21, miR-150, miR-671, miR-1249, miR-20a, miR-18b*, miR-378c, iR-4293	miR-320a, miR-320b, miR-320c, miR-769, miR-92b, miR-16	7 PD, 6 HC	[181]
Human CSF	NGS (Illumina HiSeq) + rt-qPCR, exosome	ex-miR‐126‐5p, ex-miR‐99a‐5p	–	Discovery cohort (RNA-seq): 42 PD, 43 HC; Validation cohort (rt-qPCR): 25 PD, 25 HC	[23]
	Microarray + rt-qPCR, exosome	ex-let-7c-3p, ex-miR-10a-5p, ex-miR-153, and ex-miR-409-3p	ex-miR-1, ex-miR-19b-3p	Discovery cohort: 47 PD, 27 HC; Validation cohort: 78 PD, 35 HC	[69]
	NGS (SOLiD) + rt-qPCR	miR-19a-3p, miR-19b-3p, let-7 g-3p,	**miR-10a-5p**, **miR-127-3p**, miR-128, miR-132-5p, **miR-136-3p**, miR-212-3p, miR-370, miR-409-3p, **miR-431-3p**, **miR-433**, miR-485-5p, miR-873-3p, miR-1224-5p, miR-4448	57 PD, 65 HC, (62 AD); serum + CSF; **bold** highlighted 5 miRNA are both downregulated in AD	[19]
	NGS (Illumina Nextseq 500)	let-7f-5p	miR-27a-3p, miR-423-5p	40 PD, 40 HC; miRNA as 20–24 nt	[46]
	rt-qPCR	miR-205	miR-24	28 PD, 28 HC (17 MSA)	[119]
	rt-qPCR	miR-144-5p, miR-200a-3p and miR-542-3p	–	44 PD, 42 HC	[127]
	rt-qPCR	–	miR-626	15 PD, 16 HC, 11 AD	[149]
Human Saliva	rt-qPCR	–	miR-153, miR-223	83 PD, 77 HC	[38]
	rt-qPCR	miR-145-3p, miR-874	–	30 PD, 30 HC	[32, 33]
Human Brain section	Microarray + rt-qPCR	–	miR-34b, miR-34c	11 PD, 6 controls; frontal lobe, amygdala	[126]
	rt-qPCR	–	miR-205	16 PD, 7 HC; frontal lobe; lower miR-205 cause increased LRRK2 level	[35]
	rt-qPCR	–	miR-7-5p	6 PD, 5 HC; SN	[123]
	NGS (Illumina HiSEq 2000)	–	miR-10b-5p	29 PD, 33 HC; prefrontal cortex	[79]
	Microarray	miR-22, miR-181a, miR-181b, miR-181c, miR-181d, miR-129, miR-29a, miR-29b, miR-29c, miR-373, miR-330, miR-130a, miR-130b, miR-374		6 PD, 5 HC; medulla (dorsal motor nucleus of the vagus, inferior olivary nucleus); miRNA targeted to differentially expressed genes were selected. No change of level can be obtained	[106]
	TaqMan	miR-548d	miR-198, miR-485-5p, miR-339-5p, miR-208b, miR-135b, miR-299-5p, miR-330-5p, miR-542-3p, miR-379, miR-337-5p	8 PD, 4 HC; SN	[26]
	rt-qPCR	–	miR-34b	25 PD, 26 HC; putamen; decreased miR-34b with increased adenosine A2A receptor protein level	[194]
	Microarray	miR-126	–	8 PD, 8 HC; SN-DA neurons	[90]
	rt-qPCR	–	miR-133b (midbrain)	3PD, 3HC; midbrain, (cerebral cortex, cerebellum)	[89]

#### MiRNAs in biofluids other than blood

MiRNAs can be extracted from various biofluids, including blood, serum, plasma and other sources. Since a recent study reported that the levels of miR-153 and miR-223 were decreased in the saliva of 83 PD patients versus 77 HCs, as detected by quantitative RT-qPCR [38], salivary miRNAs are now noninvasive and easy-to-access sources. Intriguingly, a Chinese study revealed increased levels of miR-874 and miR-145-3p in 30 PD patients compared to 30 HCs by RT-qPCR [33]. This discrepancy probably reflects the difference in ethnicity or technical preparation of the samples.

#### Brain-derived miRNAs in PD

Many studies extend their interest from circulating free-form miRNAs to tissue-specific miRNAs, such as brain-derived miRNAs. Circulating brain-enriched miRNAs would enable us to detect CNS signals from the peripheral bloodstream. For example, a combined group of 12 brain-enriched miRNAs, including miR-7, miR-124, miR-129, miR-139, and miR-431, could help us to differentiate individuals with PD from healthy individuals, although the discrimination using the aforementioned 4 miRNAs was moderate, with an area under the curve of 0.705 [154]. Intriguingly, Ravanidis et al. tested their 12 brain-derived miRNAs in another independent cohort with 109 idiopathic PD patients and 92 HCs [155]. This time, fewer miRNAs remained significantly different (increased miR-22-3p, miR-139-5p, miR-154-5p, miR-330-5p) between PD patients and HCs, and the highest discrimination accuracy was 0.730 by the pooled miRNAs including different miRNA candidates (miR-7-5p, miR-136-3p, and miR-409-3p). People may be curious about the accuracy of discrimination power when applying miRNA profiling in the real world. Nonetheless, we should always consider another critical issue: how can we assure that these miRNAs are definitely generated within the CNS?

#### Exosomal miRNAs in PD

Researchers have attempted to identify techniques that not only determine miRNA identities but also show the specific tissues, cells, organelles, or extracellular vesicles (EVs) from which they are derived. The characteristics of EVs, such as surface markers, size, and cargo, are extremely complicated and interwoven. Hence, the effort to categorize various EVs is an emerging field of science worldwide. By size exclusion, exosomes are one of the categories of EVs characterized by lipid bilayers and sizes of 40–160 nm [86]. Exosomes contain various cargos, including mRNAs, ncRNAs, proteins, and metabolites. The enrichment of miRNA in exosomes, abbreviated as “exo-miR” [13], is considered to participate in cell-to-cell signalling [136]. More importantly, exosomes can easily cross through the blood–brain barrier (BBB) and thus can be detected in the peripheral bloodstream and reflect homeostasis in the CNS [10].

Several studies have described the diagnostic role of exo-miR in PD [146]. Gui et al. was the first and only team to uncover a distinct pattern of exo-miRNAs in cerebrospinal fluid (CSF) of PD patients compared to HCs with decreased levels of ex-miR-1 and ex-miR-19b-3p and increased levels of ex-let-7-c-3p, ex-miR-10a-5p, ex-miR-153, and ex-miR-409-3p [69]. In a comparison with AD patients, Gui et al. also disclosed that a fraction of selected CSF-derived exo-miRNAs could be used to differentiate healthy individuals from those with PD with AD by miRNA array using RT-qPCR. Caldi et al. employed high-throughput (reads per run) small RNA sequencing by NGS to perform an unsupervised survey of CSF-derived exo-miR between PD and HC groups, and they identified another group of exo-miR, including miR-99a-5p, miR-126-5p, and miR-501-3p, as diagnostic markers of PD [23]. In a comparison of profiles of exo-miR and free-form miRNAs in CSF, there was no overlap when we examined circulating CSF-derived miRNAs, and miR-24 and miR-205 were identified as diagnostic markers separating PD patients from controls [119]. It is yet too early to determine the superiority of free-form miRNAs and exo-miRs from different biofluids in the diagnosis of PD. Major challenges also remain for selecting and extracting cell-specific exosomes (neurons, oligodendrocytes, microglia, or astrocytes in the CSF or bloodstream [78, 143].

In addition to exo-miR’s high permeability across biological membranes and blood–brain barriers, its low immunogenicity with low rejection response compared to intracranial or intravenous stem cell therapy and tissue- or cell-specific targeting ability also make it a good candidate for therapeutic druggable targets of PD. However, further research is required to confirm the therapeutic potential of exosome-based therapy in clinical applications. One of the hot topics that seems promising is using stem cell-derived exosomes. Crude exosome pellets extracted from mesenchymal stem cells (MSCs) were found to be beneficial to the outcome of traumatic brain injury [219] and cognitive deficiency [32], probably via miRNA transfer [150]. A recent study revealed that exo-miR-188-3p injection into the substantia nigra pars compacta (SNpc) of mice with 1-methyl-4-phenyl-1,2,3,6-tetrahydropyridine (MPTP)-induced PD could inhibit cell division protein kinase 5 (CDK5)-induced apoptosis and NACHT leucine-rich repeat protein 3 (NLRP3) inflammasome-induced pyroptosis [104]. These researchers transfected adipose-derived MSCs (ADSCs) with a vector containing miR-188-3p, and then, exo-miR-188-3p was abundantly secreted by ADSCs. After extraction of exo-miR-188-3p-enriched exosomes, PD-related pathogenesis, such as apoptosis and pyroptosis, could be reversed in the animal model. Nonetheless, Li et al. used intracranial injection of exo-miRs rather than the peripheral route, hindering its use in routine clinical practice. Notably, the massive generation of recombinant exosomes is still in its immature stage [61], raising questions over scientists claiming how many fractions of exosomes contain selected exo-miRs. The purity of exosomal pellets is also a major challenge because it is dramatically influenced by existing methods of isolation [92]. This issue should always be kept in mind for future experiments.

#### Pathogenic miRNAs in PD

The pathogenesis of PD comprises multiple mechanisms and can be generally classified into several categories: increased a-syn production, decreased a-syn clearance (owing to lysosome-autophagosome impairment), mitochondrial dysfunction, enhanced neuroinflammation, reduced neuronal survival, and dysregulated PD-related genes such as SNCA, PTEN-induced putative kinase 1 (PINK1), leucine-rich repeat kinase 2 (LRRK2), glucosylceramidase beta (GBA), or Parkin RBR E3 ubiquitin protein ligase (PRKN). The targeted genes of miRNAs could directly deactivate the transcription of protein-coding genes and subsequently inhibit their protein products, which is the earliest concept we know about miRNAs. For example, miR-16–1 decreases alpha-synuclein clearance by inhibiting heat shock protein 70 (hsp70) and inducing lysosomal dysfunction [232] in an experimental setting. Decreases in miR-16 or its passenger strands (miR-16–2-3p) in human leukocytes [181] or plasma [19] might be a survival strategy of neuronal cells as well as a general effect on other body tissues.

#### Neuroinflammation: miR-29c

The anti-neuroinflammatory effect of miR-29c has been extensively demonstrated in a series of studies published by Wang R. et al. in an MPTP-treated mouse model of PD and neuronal cell lines. In brief, miR-29c reduces the levels of proinflammatory interleukins (IL-1b, IL-18) [195], miR-29c-3p inhibits NLRP3 inflammasome activation by targeting nuclear factor of activated T cell 5 (NFAT5) [197] and inhibits autophagy by targeting ten-eleven translocation 2 (TET2) [200]. Several human studies have also reported consistently decreased expression of miR-29c in human serum [9, 111, 120], except one study [141] showing that miR-29c expression increased in human serum. Interestingly, no miR-29c was differentially expressed in studies using human plasma, suggesting that sample origins and the preparation protocol could make a major difference in the final list of differentially expressed miRNAs.

#### Autophagy-related alpha-synuclein clearance: let-7

The autophagosome-lysosome network is a major intracellular degradation system that digests unwanted proteins, including α-syn, in cells [225]. Genetic PD due to GBA or LRRK2 mutations presents dysregulation of intracellular vesicle transportation among autophagosomes, endosomes, and lysosomes. Aberrant let-7 miRNA levels can affect autophagy through proteins and lncRNAs. For example, increased levels of Igg-1 and atg-13 proteins can be induced by knockdown of let-7 in a *C. elegans* PD model [171]. The involvement of the mTORC1 protein pathway [48] in primary cortical neurons from transgenic mice and other lncRNAs (H19 and Lin28) in breast cancer cell lines [213] warrants further studies to elucidate the underlying mechanism between let-7 and autophagy. In humans, the let-7 family is very large [159], and different isoforms have been discovered in association studies in plasma (downregulated let-7a and let-7f expression [33]), serum (upregulated let-7d expression [11]), CSF (upregulated let-7c-3p [69] and let-7f-5p expression [46]), and CSF-derived exosomes (upregulated let-7 g-3p expression [19]) (Table 2). Determination of the most PD-relevant let-7 isoforms will require further study.

#### SNCA accumulation: miR-7 and miR-34b/c

The dysregulation of miR-7, which binds to the 3' UTR of the SNCA gene, may induce excessive α-syn generation and accumulation [47, 84]. Interestingly, two studies revealed opposite changes in the direction of miR-7 in different human sources, upregulated expression in plasma [154] and downregulated expression in the brain [123]. This kind of paradoxical phenomenon is also found in miR-34b/c, which has multiple biological functions in inhibiting SNCA transcription [85], dopaminergic neuronal survival, and mitophagy [126]. The decrease in miR-34b alone or combined with miR-34b and miR-34C is relatively consistent in postmortem findings [126, 194], albeit with its incremental change in human plasma being found only in plasma-derived exosomes (exo-miR-34b-5p actually) in one study [68] rather than free-form isoforms in numerous other studies.

**Fig. 3:**
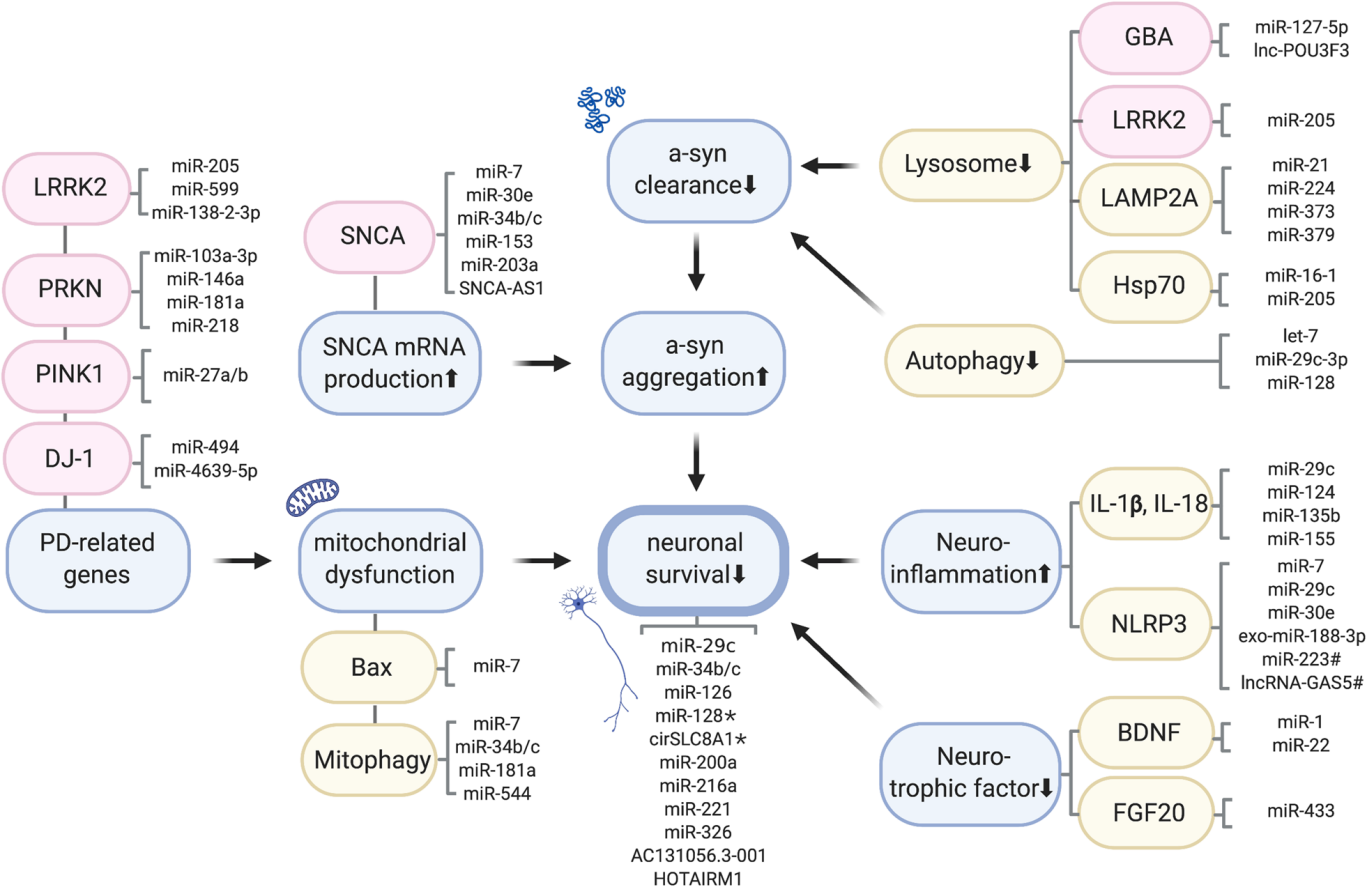
The overview of ncRNAs in the pathogenesis of PD. Several mechanisms involved in PD-relevant pathogenesis and associated ncRNAs are listed. The impairment of lysosome and autophagy systems through defects of GBA, LRRK2 genes, or LAMP2A and Hsp70 proteins reduce the clearance of α-syn. The excessive production of α-syn could be induced by hyperactive SNCA transcription associated with miRNA and lncRNA (SNCA-AS1). Aberrant α-syn metabolism results in accumulation and aggregation of phosphorylated α-syn. Several causative genes of PD such as LRRK22, PRKN, PINK1, DJ-1 are also modulated by miRNAs and largely induce mitochondrial impairment. Other groups of miRNAs also impair mitophagy or mitochondrial proteins (Bax). Together with the enhanced neuroinflammation (through pro-inflammatory cytokines and NLRP3-inflammasome) and reduced neurotrophic factors (via BDNF, FGF20), neuronal survival is damaged in the end. The interactions of miRNA-lncRNA (miR-223 and lncRNA GAS5) and miRNA-circRNA (miR-128 and cirSLC8A1) are highlighted by asterisks (*). *BDNF* Brain-derived neurotrophic factor (BDNF), *DJ-1* Protein deglycase DJ-1 / Parkinson disease protein 7; *FGF20* Fibroblast Growth Factor 20; *GBA* Glucosylceramidase Beta, *Hsp70* 70 kilodalton heat shock proteins; *LAMP2A* Lysosome-associated membrane protein 2, *LRRK2* Leucine Rich Repeat Kinase 2, *NLRP3* NLR Family Pyrin Domain Containing 3, *PINK1* PTEN-induced kinase 1, *PRKN* parkin RBR E3 ubiquitin protein ligase, *SNCA* Synuclein Alpha

The precise mechanism behind this intriguingly paradoxical phenomenon is still unclear. Taking traumatic brain injury as an example, direct release [99] or transportation via extracellular vesicles [195, 197–199] of target miRNA (miR-9 here) outside the BBB has been reported, as we found for miR-34b/c isoforms. These paradoxical biological alterations might also be relevant to patients with neurodegenerative diseases. Moreover, we should question why a decrease in miR-7 in the brain, which normally inhibits SNCA transcription, occurs in PD patients and should theoretically inhibit excessive α-syn production under normal conditions. Another study of TBI and miR-9 also suggested that altered levels of certain miRNAs in neurons might have additional benefits or unexpected harm to the growth of other types of neuronal cells, such as astrocytes [207, 208] or microglia [222]. In the near future, we need to solve the puzzle of complicated network shifts of single or multiple miRNAs in neurons and glial cells in the brain, probably with the help of advanced techniques such as single-cell RNA-seq [112]. We strongly recommend recent reviews for details of each PD-associated miRNA and related studies [132, 190]. Herein, putative miRNA- and other ncRNA-associated pathogenic pathways in PD are graphically summarized in Fig. 3.

#### MiRNA-lncRNA interactions

The modulatory property of miRNAs in the pathogenesis of PD not only have direct but also indirect effects. Another essential mechanism of indirect modulation is via the “sponge effect of lncRNA”, that is, one lncRNA can bind a large set of miRNAs and alter their activities. For example, Chen et al*.* [31] found that the lncRNA nuclear enriched abundant transcript 1 (NEAT1) could upregulate phosphodiesterase 4B (PDE4B) expression to accelerate the progression of PD by sponging miRNA-124-3p [31] and miR-374c-5p [45]. A well-known lncRNA, RNA X-inactive-specific transcript (XIST), exhibits anti-inflammatory properties by regulating the aforementioned miR-29c-3p, altering NFAT protein levels and overactivating the NLRP3 inflammasome in a rat model of epilepsy and a neuronal cell culture [229]. Another lncRNA, GAS5, demonstrated its proinflammatory effect in rotenone-induced PD mice and lipopolysaccharide (LPS)-treated microglial cells by competitively binding to miR-223-3p and then activating NLRP3 [215]. The concentration and dilution of miRNAs via lncRNAs and their sophisticated interactions are another hot topic.

### LncRNAs in PD

Studies centred on lncRNAs in PD typically emphasize several aspects, including α-syn aggregation and clearance, dopaminergic neuron degeneration, neuroinflammation, and PD-related genes, as reviewed in a recent article encompassing human, animal and cellular studies [110, 212]. In this previous review, most studies used MPTP-injected mice or neuronal SH-SY5Y cells to explore the pathogenetic role of each lncRNA interacting with these pathways. Given that one lncRNA can be multitargeted to a wide variety of downstream miRNAs, mRNAs, and PD-related genes and proteins, we expect that future studies will reveal more inspiring findings if various RNAs obtained from postmortem tissues and patient-derived dopaminergic cells are analysed in parallel [50].

#### Diagnostic lncRNAs in PD

Several human studies that used lncRNAs as diagnostic biomarkers are summarized (Table 3). Elkouris et al. identified six sense and/or antisense lncRNA genes targeting PD-related genes, including SNCA, PINK-1, UCHL-1, and MAPT, and proved their abundant existence in healthy iPSC-derived dopaminergic neurons in comparison with iPSCs and fibroblasts [50]. Next, they measured the RNA expression of six lncRNAs in the human brain from both the SN and cerebellum (9 PD and 8 HC) in human peripheral blood monocytic cells (PBMCs) (20 PD and 20 HC samples) and found that four out of six lncRNAs, *SNCA-AS1*, *MAPT-AS1*, *AK127687* and *AX747125*, were detected in CSF-derived exosomes (2 HCs). Through sophisticated experiments, Fan’s team ultimately demonstrated that these lncRNAs are enriched in dopaminergic neurons and detected in both peripheral and central tissues; thus, they are suitable as diagnostic markers of PD.

**Table 3:** The profiles of lncRNA and circRNA in PD patients

ncRNA	Samples	Analysis methods	ncRNA expression—upregulated	ncRNA expression—downregulated	Description	Ref.
lncRNA	human plasma exosome	NGS (Illumina HiSeq)	15	24/2 (2 in validation cohort: **lnc-MKRN2-42:1**, **GAS5:46**)	Discovery by NGS from 7 PD, 7 HC; validation by rt-qPCR from 24 PD, 11 HC (in **bold**)	[199]
		microarray (Aligent) + rt-qPCR	1 (Linc-POU3F3)	N.A	93 PD, 85 HC; magnetic bead isolation of exosome; only Linc-POU3F3 was highlighted. No detailed differentially expressed lncRNA profile was provided	[236]
	Human leukocytes	Microarray (Aligent)	95/4 (4 in validation cohort: AC131056.3-001, HOTAIRM1, lnc-MOK-6:1, and RF01976.1-201)	27	Discovery by microarray from 5 PD, 5 HC; validation by rt-qPCR from 72 PD and 22 HC; functional validation in SH-SY5Y and THP1 cells; AC131056.3-001 and HOTAIRM1 increases apoptosis	[52]
		Microarray (GEO database)	2 (LINC00302, LINC00328)	5 (XIST, PART1, MCF2L-AS1, NOP14-AS1, FAM215A)	50 PD, 22 HC; PRKACA, IGF1R, and lncRNA-XIST might be involved in PD pathology	[34]
		NGS (Illumina HiSeq)	38	39 (JHDM1D, LOC105378701, LOC102724104, LOC105375056, LOC105379392*)*	Discovery in 3 PD, 3 HC; validation in 2 other GEO databases; functional pairing of lncRNA and miRNA was performed and highlighted; the interaction of LOC101928100-*KLRK1*/*KLRD1* was showed but no change of level was provided)	[234]
	Human leukocytes + brain section	NGS (ABI SOLiD) + rt-qPCR	5 (**AC004744.3,** RP4-705O1.1, RP11-533O20.2, RP11-542K23.9)	8 (**RP11-79P5.3, RP13-507P19.2,** RP11-101C11.1, U1, RP11-425I13.3, RP11-124N14.3, RP11-462G22.1, PCA3)	Discovery: plasma from 3 PD, 3 HC (NGS + rt-q{CR); Validation: SN + amygdala from 6 PD and 4 HC (rt-qPCR); U1 and RP11-462G22.1 (lnc-FRG1-3) are over-expressed in PD leukocytes, and RP11-79P5.3 up-regulates in PD brains	[180]
	Human PBMCs	NGS (Illumina NextSeq 500) + rt-qPCR	1 (SCARNA2)	2 (RP1-29C18.9, RP1-29C18.8)	6 PD, 6 HC; None of lncRNAs could be validated by rt-qPCR	[58]
	Human brain + PBMCs	rt-qPCR	–	6 (***AK127687*,** *AX747125*, *** SNCA-AS1***, *UCHL1-AS1*, ***PINK1-AS1***, *MAPT-AS1*)	9 PD, 8 non-PD Controls for SN and cerebellum; lncRNA targeted to PD-related genes were enrolled; validate their existence and levels in SH-SY5Y, patients-derived iPSC, CSF-derived exosome, and human cortex	[50]
	Human brain section	Microarray data	42 (AL049437 most upregulated)	45 (AK021630 most downregulated)	11 PD, 14 HC; discovery in SN; validation by SH-SY5Y cells (tyrosine hydroxylase expression, mitochondrial mass)	[131]
		rt-qPCR	4 (lincRNA-p21, Malat1, SNHG1, TncRNA)	1 (H19 upstream conserved 1 and 2)	20 PD, 10 HC; anterior cingulate gyrus; five featured lncRNAs are PD stage-dependently expressed	[94]
circRNA	Human plasma	NGS (Illumina HiSeq X ten)	2 (SIN3A_circ_0036353; HBB_chr11:5225503–5226657: +)	9 (ITGAL_circ_0000690, SLTM_circ_0000605, YY1AP1_circ_0014606, RBM39_circ_0004870, FBXW7_circ_0001451, FAM13B_circ_0001535, RBM33_circ_0001772)	4 PD, 4 HC; testified by circRNA-miRNA-mRNA interaction network analysis	[91]
	Human PBMCs	rt-qPCR	N.A	MAPK9_circ_0001566, HOMER1_circ_0006916, SLAIN1_circ_0000497, DOP1B_circ_0001187, RESP1_circ_0004368, and PSEN1_circ_0003848	60 PD, 60 HC	[156]
	Human brain section	RNA-seq database + rt-qPCR	1 (CircSLC8A1)	N.A	Discovery from a RNA-seq database from SN of 15 PD and 10 HC; validation by rt-qPCR from SN of 24 PD and 18 HC; although 24 DE lncRNAs were mentioned in the text, details of DE lncRNA profile was not entirely clarified	[72]
piRNA	Human brain section	NGS (Illumina HiSeq) + rt-qPCR	561/46 overlapped in midbrain neurons	553/24 overlapped in midbrain neurons	8 PD, 8 HC; cingulate gyrus; the number of piRNAs overlapped in the brain tissue and midbrain neurons were highlighted	[166]

Using a microarray, Fan and his colleagues also found that four differentially expressed lncRNAs (A*C131056.3–001*, *HOTAIRM1*, *lnc-MOK-6:1*, and *RF01976.1–201*) had upregulated expression in leukocytes of PD patients [52]. The dysregulation of two lncRNAs (*AC131056.3–001* and *HOTAIRM1)* promoted apoptosis in dopaminergic neurons. Other researchers have focused on PD and other neurodegenerative diseases. Garofalo and his colleagues explored the issue by comparing the whole picture of RNA metabolism in three neurodegenerative diseases, PD, AD, and amyotrophic lateral sclerosis (ALS) [58]. High-throughput RNA-seq was exploited to identify ncRNAs in PBMCs of patients. Four lncRNAs were found to be differentially expressed in 6 PD patients versus 6 controls, with one lncRNA (SCARNA2) with upregulated expression and three with downregulated expression (2 ncRNAs: RP1-29C18.9, RP1-29C18.8; 1 protein-coding RNA: TBC1D3). Among them, only RP1-29c18.8 could not be validated by RT-qPCR in PBMCs. However, further functional and pathway analyses remain largely unexplored.

#### Exosomal lncRNAs in PD

Exosomal lncRNAs are an emerging field, as we found with miRNAs. In the plasma of 93 PD patients and 85 controls, Zou et al. isolated likely CNS-derived exosomes containing L1CAM by a microbead-based method, screened the lncRNA profile by microarray, and emphasized only one lncRNA, lnc-POU3F3, which showed highly upregulated expression in PD [236]. Although the whole picture of the differentially expressed lncRNA profile was not disclosed, the researchers highlighted a significant inverse correlation of exosomal lnc-POU3F3 levels with lysosomal enzyme β-glucocerebrosidase (GCase) activity, which is encoded by the GBA1 gene. In addition, both exosomal lnc-POU3F3 and GCase activity in PD were significantly correlated with disease severity but not exosomal α-syn levels. Therefore, this study concluded that increased exosomal lnc-POU3F3 plus decreased GCase activity in PD could serve not only as a diagnostic biomarker but also as a therapeutic target of PD.

Some studies screened for lncRNA targets by higher-throughput RNA-seq. A recent study published by Wang et al. revealed that in 7 PD patients and 7 HCs, RNA-seq identified 15 PD-relevant exosomal lncRNAs with upregulated expression and 24 with downregulated expression by ultracentrifugation of isolated exosomes (Wang Q et al. 2020). Among those differentially expressed lncRNAs, MSTRG.336210.1 and lnc‐MKRN2‐42:1 were highly expressed among controls, while MSTRG.242001.1 and MSTRG.169261.1 were highly expressed in patients. The researchers focused on motor severity-correlated lnc-MKRN2-42:1 because some of its targeted genes also had downregulated expression in the plasma of 24 PD patients and 11 HCs. Indeed, future works are still needed to validate the results in a larger cohort and clarify the biological functions of lnc-MKRN2-42:1 in animal or cell models. However, it is worth noting that Wang et al. also sequenced circRNAs and uncovered 62 circRNAs with upregulated expression and 37 with downregulated expression in their cohort, but a detailed description of these molecules is not available. We look forward to further progress in the field of lncRNAs and other ncRNAs, including circRNAs, piRNAs, and tRFs, as we will discuss in the following sections.

### CircRNAs in PD

Given that circRNAs are brain-enriched [162] and predominantly located at synapses and dendrites [226], an age-dependent accumulation of circRNAs in the CNS has been discovered in model organisms such as flies, worms, and mice [37, 203]. There are two studies focused on circRNAs in PD, one in the bloodstream and another in postmortem tissues (Table 3).

#### Blood-derived circRNAs in PD

A recent study investigated the RNA-seq profiles of circRNAs along with mRNAs and miRNAs in peripheral blood from a small number of participants, with only 4 patients with PD and 4 HCs [91]. A total of 129 circRNAs with upregulated expression and 282 with downregulated expression were found in PD samples compared to controls. Most differentially expressed circRNAs were associated with PD, AD or HD-related pathways analysed by the Kyoto Encyclopedia of Genes and Genomes. The researchers also highlighted 2 circRNAs with upregulated (chr11:5225503–5226657: + , hsa_circ_0036353) and 8 with downregulated (hsa_circ_0000690, hsa_circ_0001535, hsa_circ_0001451, hsa_circ_0004870, hsa_circ_0000605, hsa_circ_0014606, hsa_circ_0001801, hsa_circ_0001772) expression and disclosed that source genes (*HBB*, *SIN3A*, *FBXW7, ITGAL, SIN3A*) of the highlighted circRNAs were predominantly linked to functions of homeostasis and oxidative stress response, indicating the central role of reactive oxidative stress and dyshomeostasis in PD [41].

#### Brain-derived circRNAs in PD

In 2020, Hanan et al. studied circRNAs in the human brains of PD patients and examined brain tissues from three distinct regions, the amygdala, substantia nigra (SN), and mesial temporal gyrus, from 42 PD patients and 27 healthy individuals using pooled existing databases [72]. In addition to abundant mRNAs, approximately 0.02% of circRNAs were also uncovered in each sample by deep RNA-seq at 50 million reads per se. Interestingly, an age-related increase in circRNAs was similarly found in the amygdala and mesial temporal gyrus of PD patients and HCs. However, SN-derived circRNA levels tended to increase with age only in the HCs. Nevertheless, SN-derived circRNAs decreased in PD versus HC groups, which might, according to their hypothesis, be due to the loss of dopaminergic neurons in the SN of PD patients. Next, among 24 differentially expressed circRNAs found between the PD and HC groups (not clearly elaborated in the article), Hanan and her colleagues focused on a circRNA called circSLC8A1, which showed upregulated expression in the SN of PD patients. The SLC8A1 gene encodes a sodium/calcium exchanger [88]. Interestingly, miR-128-targeted mRNAs also showed upregulated expression in the SN of PD brains. A cell model was then constructed using 293HEK cells with shRNA knockdown of circSLC8A1 alone, albeit with mRNA transcripts of SLC8A1. RNA-seq eventually identified 24 out of 110 genes with upregulated expression that were targeted by miR-128 between 293HEK KO cells and controls. Taken together, the results of this study suggest that neuron-derived circSLC8A1 may modulate the functions of miR-128 and play a certain role in the pathophysiology of PD.

Another recent study utilized the most fundamental method, rt-qPCR, to screen known brain-drived circRNAs in human PBMCs [156], where they identified 48 out of 87 circRNAs. Among them, six circRNAs (MAPK9_circ_0001566, HOMER1_circ_0006916, SLAIN1_circ_0000497, DOP1B_circ_0001187, RESP1_circ_0004368, and PSEN1_circ_0003848) were significantly down-regulated in 60 PD versus 60 HC but no circRNA was found upregulated. Nevertheless, after a stepwise logistic regression selection model, there were four circRNAs with highest discrimination power between PD and HC, including SLAIN1_circ_0000497, SLAIN2_circ_0126525, ANKRD12_circ_0000826, and PSEN1_circ_0003848. Only SLAIN1_circ_0000497 and PSEN1_circ_0003848 were overlapped. Clearly, more investigation is warranted in the future.

### PiRNAs in PD

To date, only two published papers have reported their findings of specific piRNA profiles in PD based on cell and worm models, as well as postmortem tissues. The first study of piRNAs in PD was published by Schulze et al. in 2018, comparing transcriptomic and epigenomic analysis using RNA-seq between 15 lines of fibroblasts (9 PD, 6 HC), 24 lines of iPSCs (6 PD, 6 HC), and 10 lines of differentiated neurons (5 PD, 5 HC) [166]. There were no differentially expressed genes between fibroblasts, fibroblast-derived iPSCs and iPSC-differentiated midbrain neurons from the two groups, except in PD-derived neurons, where WNT3 expression was upregulated and pathways involving NOS1, CREB, and PGC1alpha were inactivated. Intriguingly, small RNA sequencing via NGS found deregulated miRNA and piRNA patterns between groups. Moreover, they replicated the protocol in the cingulate gyrus from 8 PD and 8 HC samples, aberrant expression of piRNAs, including 561 piRNAs with upregulated and 553 with downregulated expression, was also disclosed. Most targeted genomic regions were transposable elements that showed highly downregulated expression in a disease-specific manner. As a result, dysregulated piRNA features were likely due to the impact of the pathophysiology of PD itself. One more issue of concern about is the bioinformatic pipeline they used. Schulze et al. defined canonical piRNAs, or piRNA-like molecules in their context, by a nucleotide length within 24–32 bp, slightly longer than 22 bp of miRNAs, which excluded possible overlap with snoRNAs. However, many works remain to be clarified given that nearly a hundred piRNA targets await functional curation.

#### PiRNAs in a PD nematode model

Another study published by Shen et al*.* [174] used a worm model. Transgenic *C. elegans* nematodes overexpressing human α-syn wild type (WT) and the A53T mutant (HASN^WT^ OX andHASN^A53T^ OX) were crossbred with *C. elegans* with knockout (KO) of the human TDP-43-like protein *tdp-1* (*tdp-1* KO). Interestingly, among 6 various genotypes, various ncRNAs were differentially expressed between HASN^A53T^ OX and WT, including 32 miRNAs and 112 piRNAs. However, the differentially expressed ncRNAs between HASN^WT^ OX and WT only included 8 miRNAs and no piRNAs. More strikingly, a major difference in 31 miRNAs and 440 piRNAs was also uncovered when comparing HASN^WT^ OX and HASN^A53T^ OX.

#### Unknown effect of alpha-synuclein on transposon elements in PD

Apparently, there is still no convincing experimental evidence demonstrating that WT and A53T α-syn have diverse impacts in influencing TEs through distinct piRNA alterations. However, the dramatic change of piRNAs in A53T mutant nematodes suggests that maybe it is mutant strain rather wild type α-syn that should be investigated in relation to the piRNA—TE loop deregulation. Another misfolded protein commonly associated with neurodegenerative disease, tau, is a hot topic in studying tau-depleted piRNA and dysregulated TE patterns in AD [71] and tauopathies [187]. More studies to discover the interplay between the α-syn-piRNA-TE axis are warranted in the near future.

### tRNA fragments in PD

A recent review summarizing the discovery of tRFs in neurodegenerative diseases, including PD, is available [148]. Unfortunately, there is only one human study that revealed altered tRF patterns in patients with PD [113]. Three existing RNA-seq samples from the prefrontal cortex, CSF, and serum of PD patients and controls were collected and reanalysed to identify differentially expressed tRFs between groups. The discrimination of patients and healthy subjects by selective tRF profiles further yielded a high sensitivity and specificity despite sex-dependent tRF expression. These preliminary findings warrant more validation studies with a larger sample size or more types of parkinsonism syndromes to determine the true power of tRFs in the differential diagnosis of PD.

Another study focused on brain-derived tRFs in mice called senescence-accelerated mouse prone 8 (SAMP8) [231]. Intriguingly, tRFs with a miRNA-like pattern were found to primarily target a causative gene of PD, *Park2,* or *Parkin*. In 43 PD-related PARK families, autosomal-recessive early onset PD induced by *Parkin* mutation is the most common genetic cause of familial PD worldwide [75]. However, SAMP8 mice are typically not considered a model of PD but ageing or early AD [142], characterized by autophagic deficits, mitochondrial dysfunction, excessive oxidative stress, and, most importantly, tau protein aggregation. It would be more convincing for us to determine the pathoetiology of tRFs in PD if similar findings were replicated in transgenic mouse models of PD.

#### Summary

The discovery of diagnosis, treatment, prognosis-related ncRNAs in PD is challenging, largely owing to the complexity of disease itself, the variability of sample origins (blood-, brain-, urine-, saliva-, or exosome-derived), the non-standardized method using distinct RNA-seq platforms and software pipelines, and inconsistent results of ncRNA candidates in human association studies. Validations of robust ncRNAs relevant to the differential diagnosis between PD, HC or disease controls, and disease progression in motor, psychiatric, cognitive domains should be further clarified with the highest interest. To date, only a short list of ncRNA candidates can be replicated in mechanisms-based experiments using cell or animal models. Most of pathogenic miRNAs in PD which also have significant alteration in human studies were highlighted as aforementioned. Discoveries of safer virus- [17] and novel exosome-based [114] delivery platform associated with highly efficient knockdown system [74] are increasing, mostly related to the use of miRNA [12] and circRNA [8]. Briefly, ncRNA candidates are more promising in the translation from disease-relevant biomarkers to treatment-based druggable targets [36]. The advance in RNA-based therapy from the recent experience of mRNA-based COVID vaccines can certainly make a giant step forward to the development of ncRNA-based disease-modifying treatments in the future.

## Concluding remarks

The discovery of novel ncRNAs with diverse biological functions within different species and cell types makes ncRNAs one of the most exciting scientific topics. In this review, we have introduced many ncRNAs, including miRNAs, lncRNAs, piRNAs, circRNAs, and tRFs. MicroRNAs, lncRNAs and circRNAs can serve as diagnostic biomarkers, while miRNAs are the most promising therapeutic targets of PD, especially coupled with exosomal transportation. However, the evidence for piRNAs and tRFs is less convincing at this stage.

There are still many new classes of ncRNA sequences to be found by higher-throughput, longer-read, and extensive sequencing platforms. A complementary approach using multiple platforms is often recommended when generating RNA profiles or analysing RNA expression. However, their biological functions still warrant further clarification by functional analysis in the pathophysiology of PD.

The complex network of ncRNAs with DNA, proteins, and other ncRNAs makes this puzzle hard to unravel. With the help of collaborative sequencing and analytical methods and proper selection of model organisms, we can expect to gain clearer insight to identify disease-relevant ncRNAs as diagnostic biomarkers or therapeutic targets of PD in all biological aspects in the near future.

## Supplementary Information

**Additional file 1. Appendix.** Pros and cons of RNA sequencing platforms.

## Data Availability

Not applicable.

## Abbreviations

ABS: Alternative back-splicing
AD: Alzhimer’s disease
ADAR: Adenosine deaminase
ADSC: Adipose-derived MSCs
AGO: Argonaute
AGO2: Argonaute 2
ALS: Amyotrophic lateral sclerosis
BBB: Blood–brain barrier
CBD: Corticobasal degeneration
CCS: Circular consensus sequence
CDK5: Cell division protein kinase 5
CDR 1: Cerebellar degeneration-related protein 1
circRNA: Circular RNA
CNS: Central nervous system
Cq: Quantitative cycle
DGCR8: DiGeorge syndrome critical region 8
DLB: Dementia with Lewy bodies
eRNA: Enhancer RNA
exo-miR: MiRNA in exosomes
GCase: β-Glucocerebrosidase
GBA: Glucosylceramidase Beta
HD: Huntington disease
hsp70: Heat shock protein 70
iPSC: Induced pluripotent cell
LB: Lewy bodies
lncRNA: Long non-coding RNA
LRRK2: Leucine-rich repeat kinase 2
MID: The middle domain
miRISC: MiRNA-induced silencing complex
miRNA: MicroRNA
mRNA: Messenger RNA
MSA: Multiple system atrophy
MSC: Mesenchymal stem cell
ncRNA: Non-coding RNA
NEAT1: Nuclear enriched abundant transcript 1
NLRP3: NAcht Leucine-rich repeat protein 3
nt: Nucleotides
ONT: Oxford Nanopore Technologies
ORF: Open reading frame
lncRNA: Long non-coding RNA
PAZ: Piwi–Argonaute–Zwille domain
PBMC: Peripheral blood monocytic cell
PD: Parkinson disease
PDE4B: Phosphodiesterase 4B
PINK1: PTEN-induced putative kinase 1
piRNA: PIWI-interacting RNA
PIWI: The P-element induced wimpy testes domain
Pol II: RNA polymerase II
pre-miRNA: Precursor microRNA
pre-tRNA: Precursor tRNA
pri-miRNA: Primary microRNA
PRKN: Parkin RBR E3 Ubiquitin Protein Ligase
PSP: Progressive supranuclear palsy
qPCR: Real-time quantitative PCRs
RBP: RNA binding proteins
ROI: Read of insert
rRNA: Ribosomal RNA
tRNA: Transfer RNA
RT: Reverse transcription
sitRNA-5: Stress-induced tRNA-5
SN: Substantia nigra
SNpc: Substantia nigra pars compacta
TE: Transposon element
TF: Transcription factor
tiRNA: TRNA halves
tRF: TRNA-derived fragment
tRNA: Transfer RNA
trRNA: TRNA-derived small RNAs
UMI: Unique molecular identifiers
WT: Wild type
α-syn: Alpha-synuclein
