# Supplementary material for: The role of noncoding RNAs in Parkinson’s disease: biomarkers and associations with pathogenic pathways
Source: J Biomed Sci. 2021 Nov 18;28:78. doi: 10.1186/s12929-021-00775-x (PMC8603508; doi:10.1186/s12929-021-00775-x)
Supplement: Supplementary file 1 — Additional file 1. Appendix. Pros and cons of RNA sequencing platforms. [file 12929_2021_775_MOESM1_ESM.docx]

**Additional file 1**

**Appendix. Pros and cons of RNA sequencing platforms**

**Microarray**

Pros

- Good for fast transcriptome profiling; can simultaneously provide the expression levels of hundreds of target RNA templates (Bumgarner et al., 2013)
- Can analyze transcripts with different sizes (both small and long RNAs, fragmentation is not required)

Cons

- Pre-signed probes are necessary, thus cannot analyze or identify novel transcripts. Not suitable for RNA sequencing
- Potential probe binding defects (e.g. cross-hybridization, non-specific hybridization) may occur during the reaction
- Specificity and sensitivity can be compromised in small RNAs with high sequence similarity as well due to the nature of short hybridization sequences (Siddika & Heinemann, 2021)
- There are also issues associated with probe redundancy and annotation (Zhao et al., 2014)

**RT-qPCR**

Pros

- Very sensitive to the quantitative differences, suitable for expression analysis
- Currently the gold standard method to verify data obtained by microarrays or NGS approaches (Siddika & Heinemann, 2021)
- Can analyze transcripts with different sizes (both small and long RNAs, fragmentation is not required)

Cons

- Potential primer binding defects may occur during the reactions
- Can only run a limited number of reactions at a time depending on the qPCR plate
- Pre-signed primers are necessary, thus cannot analyze or identify novel transcripts.  Not suitable for sequencing

**NGS**

Pros

- Higher throughput (reads per run) than long-read platforms
- High sequencing volume; can sequence millions of fragments (hundreds to thousands of transcripts) simultaneously in parallel
- Accurate data acquisition, good sequence coverage and depths
- Suitable for minor variant detections and de novo transcript discovery

Cons

- Expression analysis based on counts of mapped reads (statistical correction is required)
- Fragmentation is necessary for long transcripts (>500 bp), thus not suitable for isoform / splice form studies
- Data analyses, e.g. alignment, transcriptome reconstruction, read counts, and expression analysis, require a large amount of computer algorithm (table 2)

**PacBio**

Pros

- Good for isoform / splice form analysis
- Can sequence full-length transcript (fragmentation is not required)
- Can generate higher quality raw data with a lower error rate and higher mappability compared to ONT raw data (Weirather et al., 2017)

Cons

- Weak power in detecting short RNA sequences or degraded RNA templates. Short read lengths (<500 bp) had low alignment rates
- Higher sequencing error rate than NGS
- Expression analysis based on counts of mapped reads (statistical correction is required)

**Nanopore**

Pros

- Good for isoform / splice form analysis
- Can sequence full-length transcript (fragmentation is not required)
- PCR amplification is not required, avoiding potential PCR errors
- Compared to PacBio sequencing, one advantages of ONT is that it can estimate transcript expression levels (Cui et al., 2020)

Cons

- Weak power in detecting short RNA sequences or degraded RNA templates. Short read lengths (<500 bp) had low alignment rates
- Higher sequencing error rate than NGS and PacBio (Weirather et al., 2017)
